# Rectus Sheath Hematoma Following Cesarean Section: A Concealed Extrauterine Cause of Postpartum Hemorrhage

**DOI:** 10.7759/cureus.108664

**Published:** 2026-05-11

**Authors:** Bhavna Kumre, Nikita Vijay, Sheela Jain, Nalini Humaney

**Affiliations:** 1 Department of Obstetrics and Gynecology, N. K. P. Salve Institute of Medical Sciences and Research Centre and Lata Mangeshkar Hospital, Nagpur, IND; 2 Department of Medicine, N. K. P. Salve Institute of Medical Sciences and Research Centre and Lata Mangeshkar Hospital, Nagpur, IND

**Keywords:** acute kidney injury, cesarean, concealed postpartum hemorrhage, hemodialysis, rectus sheath hematoma, severe pre-eclampsia

## Abstract

Rectus sheath hematoma (RSH) is a rare but potentially life-threatening extrauterine cause of postpartum hemorrhage, particularly following cesarean delivery in high-risk patients, like those with severe preeclampsia. It usually results from injury to the epigastric vessels or rectus muscle and may be aggravated by preeclampsia-related endothelial dysfunction. Because its presentation can mimic common postoperative or postpartum complications, diagnosis is often delayed. Severe cases that go unnoticed or are missed result in concealed bleeding, shock, organ failure, or death. A 30-year-old primigravida underwent an emergency cesarean delivery at term for severe preeclampsia and subsequently developed abdominal distension, acute anemia with hypovolemic shock, and acute kidney injury in the immediate postoperative period. Initial ultrasonography was non-diagnostic; however, repeat imaging identified a significant collection beneath the rectus sheath, which was confirmed intraoperatively as an RSH. The patient was managed with aggressive volume resuscitation, surgical evacuation of the hematoma, and hemodialysis support, following which she recovered clinically. This case emphasizes the need to consider RSH in post-cesarean patients with unexplained postpartum hemorrhage, abdominal pain or distension, hemodynamic instability, and falling hemoglobin levels in the absence of an identifiable uterine or genital source of bleeding. The report also acknowledges the benefits and limitations of ultrasonography and emphasizes the importance of early recognition, prompt imaging evaluation, and multidisciplinary management in optimizing maternal outcomes.

## Introduction

Postpartum hemorrhage (PPH), defined by the International Federation of Gynecology and Obstetrics (FIGO) as blood loss exceeding 1000 mL following cesarean delivery within 24 hours after birth or any blood loss sufficient to compromise hemodynamic stability, remains a leading cause of maternal morbidity and mortality worldwide, accounting for nearly 25-30% of maternal deaths globally [1]. Current guidelines, including those from FIGO (2022) and the American College of Obstetricians and Gynecologists (Practice Bulletin No. 183), primarily focus on uterine and genital tract causes of PPH, commonly summarized by the “four Ts”: tone, trauma, tissue, and thrombin [1,2]. However, extrauterine or extragenital sources of concealed hemorrhage, including rectus sheath hematoma (RSH), remain under-emphasized in standard diagnostic algorithms, potentially contributing to delayed recognition and increased maternal morbidity [3-6].

RSH is an uncommon clinical entity, accounting for approximately 1-2% of acute abdominal pain cases in the general population [3]. Nonetheless, its actual prevalence among obstetric patients, especially in the postpartum phase, remains ambiguous and is probably underreported. RSH results from rupture of the epigastric vessels or their branches supplying the anterior abdominal wall or from direct tearing of rectus muscle fibers, often precipitated by surgical trauma, anticoagulant therapy, or sudden increases in intra-abdominal pressure due to coughing or vomiting [3]. Anatomically, the inferior epigastric vessels are particularly vulnerable during cesarean delivery because the posterior rectus sheath is absent below the arcuate line, located approximately midway between the umbilicus and pubic symphysis [3]. Recent case reports and case series have reported postpartum RSH in women with hypertensive disorders of pregnancy, including severe preeclampsia [5-8]. Although the exact relationship remains unclear, preeclampsia-associated endothelial dysfunction and vascular fragility may potentially predispose to hemorrhagic complications.

Clinically, RSH may present postpartum with abdominal pain, abdominal distension, a palpable abdominal mass, tachycardia, hypotension, and declining hemoglobin levels-features that may closely mimic uterine or genital tract causes of PPH and contribute to diagnostic confusion [6,9]. Clinical clues suggestive of an extrauterine source include a well-contracted uterus with minimal vaginal bleeding despite ongoing hemodynamic instability, persistent wound-site ooze, or progressive abdominal distension. Delayed diagnosis may result in severe complications, including hypovolemic shock, coagulopathy, end-organ dysfunction, sepsis, and death [6,9-12]. Although ultrasonography and CT can aid diagnosis, imaging may be challenging in hemodynamically unstable postpartum patients.

We report a critical case of a postnatal woman with severe preeclampsia who developed a life-threatening RSH following cesarean delivery, resulting in massive PPH, hypovolemic shock, and subsequent acute kidney injury (AKI). Reports of RSH presenting as severe PPH and complicated by AKI in the immediate post-cesarean period are exceedingly rare [9,10], highlighting an important gap in the current obstetric literature. This case underscores the diagnostic challenges posed by concealed extrauterine hemorrhage, emphasizes the potential pathophysiologic interplay between severe preeclampsia and RSH, and highlights the importance of early recognition and multidisciplinary management, including prompt surgical intervention and renal support. This report was prepared in accordance with the CARE (CAse REport) guidelines [13].

## Case presentation

A 30-year-old primigravida was referred to our tertiary care center on postoperative day two following an emergency lower-segment cesarean section (LSCS) performed for severe preeclampsia with breech presentation. She presented with progressive dyspnea, persistent serosanguinous wound discharge, abdominal distension, oliguria, and severe anemia, raising suspicion of concealed PPH with associated AKI and possible coagulopathy.

Her antenatal course was complicated by gestational hypertension diagnosed at 32 weeks of gestation (blood pressure: 140/98 mmHg), initially controlled with oral labetalol (100 mg twice daily). At 36 weeks, worsening hypertension associated with generalized edema required escalation of antihypertensive therapy with labetalol and nifedipine (10 mg three times daily). At 37 weeks, she developed blurred vision, headache, severe hypertension (180/120 mmHg), and 3+ proteinuria, consistent with severe preeclampsia, necessitating emergency LSCS under spinal anesthesia. Preoperative laboratory investigations demonstrated mild anemia with preserved renal, hepatic, and coagulation parameters (serum creatinine: 0.7 mg/dL; PT/INR: 13 sec/0.8; aPTT: 26 sec) (Table 1). The intraoperative and immediate postoperative periods were uneventful.

**Table 1:** Key investigations and serial laboratory trends during hospital course Abbreviations: ALT, alanine aminotransferase; AST, aspartate aminotransferase; BUN, blood urea nitrogen; LDH, lactate dehydrogenase; LSCS, lower segment cesarean section; POD, postoperative day; TLC, total leukocyte count POD2A: Before exploratory laparotomy; POD2B: After exploratory laparotomy

Parameter	Reference Range	Pre-LSCS	POD 1	POD 2A	POD 2B	POD 3	POD 5	POD 6	POD 8	POD 10	POD 15
Hemoglobin (g/dL)	11-13	9.2	6.7	7.4	7.6	-	-	8.1	-	9	9.2
Hematocrit (%)	25-40	26	19	19.5	20.2	-	-	21	-	22	26
Platelets (×10⁹/L)	150-400	176	160	150	165	-	-	162	-	170	176
TLC (×10⁹/L)	12-20	13	61	43	46	-	-	28	-	20	13
Lactate (mmol/L)	<2	-	-	1.9	1.8	-	-	-	-	-	-
Creatinine (mg/dL)	0.4-0.8	0.8	2.32	2.36	4.3	5.2	3.44	5.34	5.94	4.74	1.33
BUN (mg/dL)	3-11	10	79	80	96	100	78	90	96	48	20
Sodium (mmol/L)	130-140	-	135	143	134	133	136	134	-	140	136
Potassium (mmol/L)	3.5-5.0	-	3.6	4.27	4.7	4.8	4.9	5.0	-	3.44	3.5
Bilirubin (mg/dL)	0.1-1.2	0.2	0.3	0.3	-	0.4	-	-	-	0.2	-
ALT (U/L)	10-35	35	36	35	-	38	-	-	-	36	-
AST (U/L)	10-40	40	42	46	-	47	-	-	-	44	-
LDH (IU/L)	220-400	-	-	257	-	300	-	-	-	-	-
pH	7.40-7.47	-	-	7.40	7.30	7.33	-	7.40	7.41	-	-
pCO₂ (mmHg)	27-32	-	-	32	36	34	-	33	32	-	-
pO₂ (mmHg)	100-105	-	-	96	90	89	-	95	96	-	-
HCO₃⁻ (mmol/L)	18-22	-	-	21.8	22	21	-	23	22	-	-

Approximately six hours postoperatively, serosanguinous wound discharge was noted. Initial ultrasonography at the referring hospital excluded intra-abdominal and genital tract bleeding, demonstrating a well-contracted postpartum uterus without intrauterine or intraperitoneal collection. Hemostatic skin and subcutaneous sutures were placed under sedation with temporary improvement. However, by 12 hours postoperatively, wound discharge recurred and was accompanied by worsening dyspnea, tachycardia, oliguria (50 mL/12 h), severe anemia (hemoglobin: 6.2 g/dL; hematocrit: 19%), leukocytosis (61 × 10⁹/L), and rising serum creatinine (2.32 mg/dL). Although the blood pressure reported was 120/70 mmHg, the value indicated hemodynamic decompensation relative to her previous severe hypertension. Due to progressive clinical deterioration, she was referred to our institution for multidisciplinary management. The chronological clinical course is summarized in Table 2.

**Table 2:** Timeline of clinical course during hospitalization Abbreviations: AKI, acute kidney injury; ATN, acute tubular necrosis; BUN, blood urea nitrogen; Cr, creatinine; FFP, fresh frozen plasma; I/O, input/output; KDIGO, Kidney Disease: Improving Global Outcomes; POD, postoperative day; POCUS, point-of-care ultrasonography; PRC, packed red cells; UO, urine output

Phase	Time Point	Renal Parameters (Cr and BUN mg/dL) and Fluid Balance	Clinical Course
Antepartum	32-37 weeks	-	Gestational hypertension progressed to severe preeclampsia
Delivery	Day 0	Cr: 0.8 mg/dL; BUN: 10 mg/dL	Emergency cesarean delivery for severe preeclampsia with breech presentation; 2140 g neonate delivered; no intraoperative hemorrhage
Early Postoperative Period	POD 1	UO 50 mL/12 h; rising Cr: 2.3 BUN: 49 mg/dL	Surgical site bleeding (at 6 hours) with recurrent serosanguinous discharge and hemodynamic deterioration (at 12 hours). Significant fall in hemoglobin and hematocrit. Intra-abdominal, uterine, and genital tract bleeding excluded. Referral to tertiary facility
Tertiary Care Admission	POD 2	Persistent oliguria: UO 50 mL/12 h	Shock index 1.15; POCUS showed ~450 mL sub-rectus collection; resuscitated with crystalloids, antibiotics, 3 PRC, 4 FFP
Surgical Intervention	POD 2	-	Exploratory laparotomy confirmed type II rectus sheath hematoma; hematoma evacuated, the bleeding vessel ligated; subrectus drain placed. Intra-operative blood loss: 150-200 mL
ICU Course	POD 2-3	UO remained 50 mL/12 h	Persistent oliguria despite fluids, transfusion (2 PRC, 2 platelet units), and diuretics
AKI Management	POD 3	I/O 2650/75 mL; Cr: 5.2 mg/dL	KDIGO stage 3 AKI secondary to ischemic ATN; first hemodialysis
	POD 4	I/O 2500/500 mL	Second hemodialysis; transfused 1 PRC
	POD 5	I/O 2500/800 mL; Cr: 3.44 mg/dL	Partial renal recovery
	POD 6	I/O 2500/300 mL; Cr: 5.9 mg/dL	Third hemodialysis; oral furosemide initiated
Recovery	POD 7-14	UO improved to 700-2300 mL/day. POD 10: Cr 4.7 mg/dL	Clinical stabilization; fourth hemodialysis on POD 8
Discharge	POD 15	I/O 2300/2000 mL, Cr 1.3 mg/dL	Discharged stable with controlled BP and healed wound
Follow-Up	POD 30	Cr: 0.7 mg/dL; BUN: 16 mg/dL	Normotensive off antihypertensives; wound healed

On admission to the intensive care unit, the patient was alert, afebrile, and pale, with facial puffiness, pedal edema, tachycardia (139 bpm), tachypnea (22-24 breaths/min), and a blood pressure of 120/70 mmHg. The shock index was elevated at 1.15, suggestive of hemodynamic compromise. There was 30 mL of concentrated urine in the urobag. Abdominal examination revealed distension, wall edema, and guarding without a discrete palpable mass or skin discoloration. Persistent serosanguinous discharge was present from the surgical wound. Due to severe postoperative pain and guarding, Carnett’s and Fothergill’s signs - used to distinguish abdominal wall from intra-abdominal pathology - were not assessed. Pelvic examination demonstrated vulval edema, minimal vaginal bleeding, a patulous cervix, and a well-contracted uterus without evidence of genital tract hematoma.

Repeat laboratory investigations confirmed severe anemia (hemoglobin: 7.4 g/dL; hematocrit: 19.5%) with preserved platelet count and coagulation profile (prothrombin time/international normalized ratio (PT/INR): 13 sec/0.9; activated partial thromboplastin time (aPTT): 26 sec). Liver enzymes remained normal, while renal function was impaired (serum creatinine: 2.36 mg/dL; blood urea: 80 mg/dL), indicating evolving AKI (Table 1). Although the exact blood loss was difficult to estimate, significant hemorrhage was inferred from marked postoperative hemoglobin decline (1 g/dL Hb drop ≈ 450-500 mL blood loss) and concurrent hemodynamic deterioration. The estimated blood loss was approximately 1.2-1.3 L. Marked leukocytosis prompted evaluation for sepsis; however, serum lactate remained normal (1.9 mmol/L), and subsequent blood and urine cultures were sterile. The leukocytosis was interpreted as a severe stress response secondary to major hemorrhage and surgical stress after infectious etiologies were excluded.

Bedside point-of-care ultrasonography (POCUS) using a portable ultrasound system (Samsung HM70 EVO, Samsung Medison Co., Ltd., Seoul, South Korea) with a low-frequency curvilinear transabdominal probe (2-5 MHz) in longitudinal and transverse views demonstrated an approximately 450 mL heterogeneous, predominantly hypoechoic collection within the rectus sheath at the incision site, without intraperitoneal extension. The uterine cavity appeared empty, with minimal free fluid in the peritoneal cavity, and color Doppler imaging demonstrated no internal vascular flow within the collection. Although non-contrast CT was considered, imaging was deferred because of the patient’s hemodynamic instability, evolving AKI, and the immediate availability of surgical intervention. Contrast-enhanced imaging and angiography were avoided due to concern for contrast-induced nephropathy in the setting of pre-existing renal dysfunction.

Massive hemorrhage protocol was initiated with the transfusion of three units of packed red blood cells (300 mL each), four units of fresh frozen plasma (250 mL each), and two units of platelets (50 mL) preoperatively. Empirical broad-spectrum antibiotics (meropenem and metronidazole) were administered while awaiting culture reports because occult sepsis could not initially be excluded. Following multidisciplinary evaluation involving obstetricians, intensivists, anesthesiologists, nephrologists, and surgeons, emergency surgical exploration laparotomy was performed under general anesthesia after obtaining high-risk informed consent.

**Figure 1:**
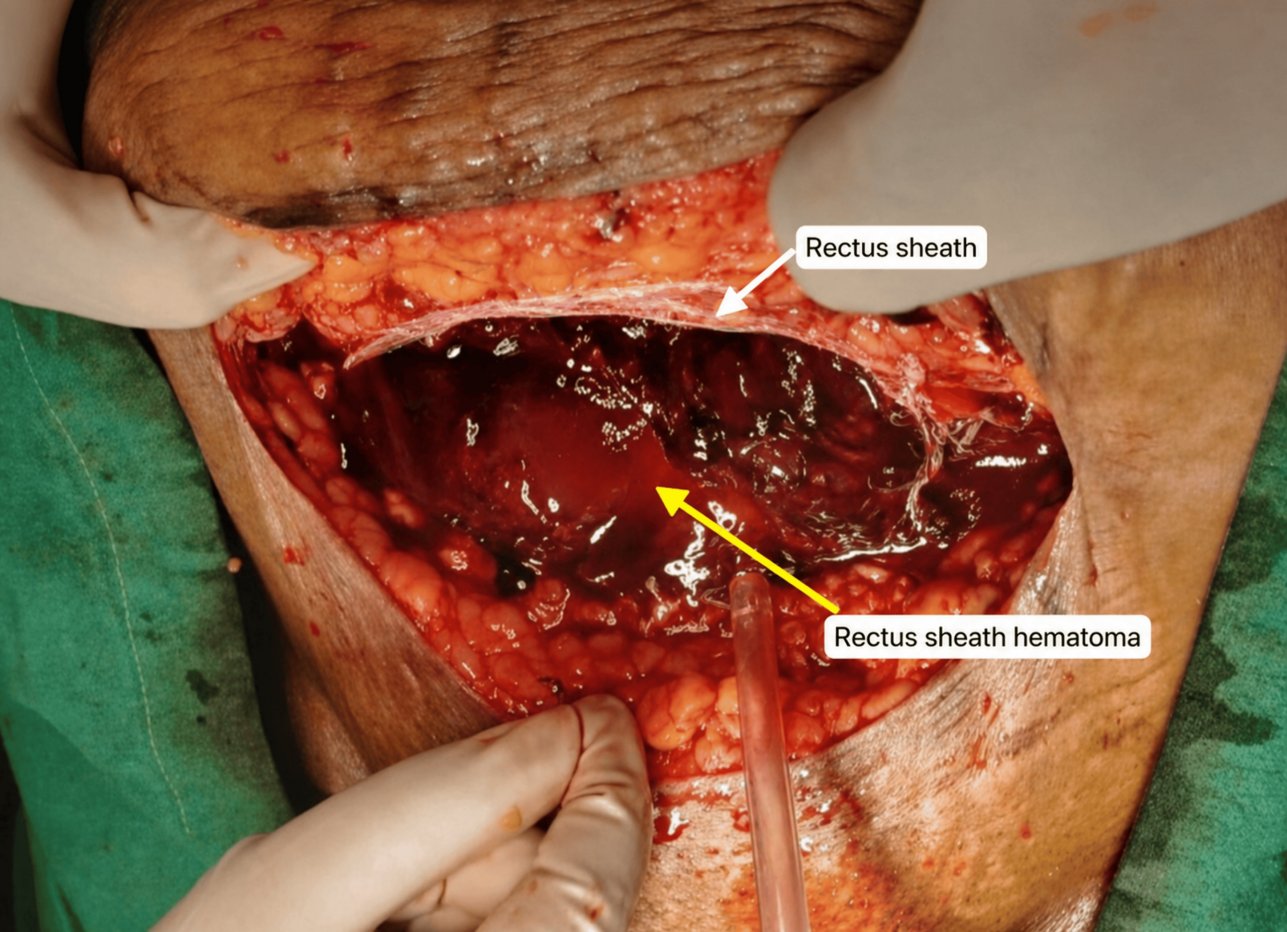
Intraoperative view demonstrating rectus sheath hematoma during exploratory laparotomy Following a Pfannenstiel incision and opening of the anterior rectus sheath (white arrow), a large hematoma (yellow arrow) was identified beneath the rectus sheath. A suction cannula is seen evacuating the collection. Orientation: cranial (top), caudal (bottom). Scale: surgical field view.

Intraoperatively, reopening of the previous incision and anterior rectus sheath revealed a large 15 × 8 cm hematoma located beneath the rectus sheath and anterior to the parietal peritoneum, with no intraperitoneal extension (Figure 1). Cesarean sutures over the lower uterine segment were intact, and the uterus was well contracted. Following hematoma evacuation, active bleeding from a branch of the right superior epigastric artery was identified and ligated. Hemostasis was secured, a sub-rectus drain was placed, and the abdomen was closed in layers.

Postoperatively, the patient required intensive multidisciplinary management for hemodynamic stabilization, blood pressure control, renal support for AKI, and surveillance for infectious and cardiopulmonary complications. Two additional units of packed red blood cells were transfused postoperatively. Hypertension was managed with intravenous labetalol and oral nifedipine. Oxygen saturation remained above 96% on room air, with no evidence of pulmonary edema or respiratory compromise. Hemodynamic status improved significantly after transfusion and fluid resuscitation, with a reduction of the shock index from 1.15 to 0.5. Subsequent laboratory investigations demonstrated improved hematologic and coagulation parameters with stable liver function tests (Table 1).

Despite adequate fluid resuscitation, transfusion support, and diuretic challenge with furosemide, oliguria persisted with progressive azotemia and mild metabolic acidosis (pH 7.30). Serum electrolytes and electrocardiography remained normal. An elevated central venous pressure (12 mmHg; normal range 3-6 mmHg) with a normal bedside echocardiography ruled out cardiogenic hypoperfusion and supported intrinsic renal injury. AKI was considered multifactorial, predominantly secondary to hemorrhagic hypoperfusion superimposed on severe preeclampsia-associated renal endothelial dysfunction, resulting in ischemic acute tubular necrosis. Based on a more than threefold rise in serum creatinine from baseline, the patient fulfilled Kidney Disease: Improving Global Outcomes (KDIGO) stage 3 AKI criteria. Due to persistent renal dysfunction and concern regarding fluid overload with increased risk of pulmonary edema, hemodialysis was initiated.

The patient subsequently underwent four sessions of hemodialysis and received one additional unit of packed red blood cells. Progressive improvement in urine output and renal function was observed thereafter, with serum creatinine declining to 1.3 mg/dL by postoperative day 15. Follow-up ultrasonography demonstrated no residual collection, and drain output progressively decreased, permitting drain removal on postoperative day four. Blood and urine cultures remained sterile, leukocyte counts normalized, and antibiotics were discontinued appropriately. The patient showed steady clinical recovery with normalization of urine output, improved renal parameters, controlled blood pressure, satisfactory wound healing, and recovery of hemoglobin levels. She was discharged on postoperative day 15 in stable condition. At follow-up, she remained asymptomatic with complete wound healing, normal renal function, and controlled blood pressure (120/80 mmHg). The chronological timeline of the patient’s clinical course, investigations, interventions, and recovery is summarized in Table 2.

## Discussion

RSH is a rare but potentially life-threatening extrauterine cause of concealed PPH following cesarean delivery. Because its clinical presentation frequently overlaps with more common obstetric and postoperative complications, including uterine atony, intra-abdominal hemorrhage, paralytic ileus, and surgical site infection, diagnosis is often delayed [3,8]. In the present case, the initial assessment focused primarily on common causes of PPH and preeclampsia-related complications, while the evolving abdominal wall hematoma remained unrecognized. This delay contributed to the development of significant concealed hemorrhage, hemodynamic instability, and severe AKI. Our case highlights the importance of considering RSH in postpartum women presenting with unexplained anemia, oliguria, abdominal distension, wound ooze, and hemodynamic instability despite a well-contracted uterus and minimal vaginal bleeding.

Although the precise incidence of post-cesarean RSH remains uncertain because of underreporting, increasing cesarean delivery rates, wider use of anticoagulants, and improved imaging availability have likely contributed to increased recognition [6,14]. Several case reports and case series have documented RSH following cesarean delivery [5-10,12,14-17]; however, severe presentations complicated by hypovolemic shock and dialysis-requiring AKI remain uncommon [8,9]. Chandran et al. reported AKI requiring hemodialysis in two patients with severe post-cesarean RSH requiring relaparotomy [9], while Agrawal et al. described a fatal case complicated by multiorgan dysfunction syndrome and coagulopathy [8]. These reports emphasize that delayed recognition of RSH may result in substantial maternal morbidity and mortality.

RSH most commonly results from injury to the superior or inferior epigastric vessels or tearing of the rectus muscle fibers [3]. In cesarean delivery, excessive tissue retraction, lateral dissection, inadequate hemostasis, or suture placement near the rectus borders may predispose to vascular injury [4,5]. Additional risk factors include anticoagulant use, coughing, previous abdominal surgery, and coagulation abnormalities [6-9]. In our patient, severe preeclampsia may have further contributed through endothelial dysfunction, severe hypertension, and impaired vascular integrity. Several published postpartum RSH cases have similarly occurred in women with severe preeclampsia or hypertensive disorders of pregnancy [5-8]. However, the association between preeclampsia and increased RSH risk remains speculative and requires further investigation.

The coexistence of severe preeclampsia and concealed hemorrhage likely contributed to the severity of renal dysfunction in our patient. A major RSH that accompanies severe preeclampsia elevates the risk of AKI due to common pathological mechanisms [18]. Significant blood loss can precipitate renal hypoperfusion and ischemic acute tubular necrosis, as evidenced in documented cases [8,9,18]. Severe preeclampsia independently predisposes patients to renal endothelial injury, vasospasm, and reduced autoregulatory reserve [18]. Consequently, even a modest amount of bleeding in such patients may rapidly precipitate severe AKI. Our patient fulfilled KDIGO stage 3 AKI criteria and required temporary hemodialysis despite definitive surgical management and aggressive resuscitation [19]. This underscores the importance of early recognition, serial renal monitoring, and timely nephrology involvement in high-risk obstetric patients.

Clinical diagnosis of RSH in the immediate postoperative period is challenging because classical bedside signs such as Carnett’s and Fothergill’s signs are often difficult to elicit due to postoperative pain, guarding, and restricted mobility [3]. Similarly, Cullen’s and Grey Turner’s signs are late manifestations and have limited value in early diagnosis [3]. In our patient, the absence of significant vaginal bleeding despite progressive anemia and shock, together with a well-contracted uterus, raised suspicion for an occult extrauterine source of hemorrhage and prompted urgent laboratory evaluation and bedside imaging.

Prompt lab tests - including CBC, coagulation profile, liver function, and renal tests - are crucial to gauge hemorrhage severity, detect coagulopathy, assess organ perfusion, and guide diagnostic and therapeutic interventions. Postpartum RSH cases have shown rapid hemoglobin drop, coagulation failure, and renal impairment, indicating possible multisystem effects and serious maternal morbidity [5,7-8,12-13,15-16,18]. These lab results enable targeted transfusion, correct coagulopathy, and support interventional procedures.

Imaging plays a key role in diagnosis. CT, including non-contrast and contrast-enhanced angiography imaging, is considered the gold standard for diagnosing RSH because of its high sensitivity, ability to delineate hematoma extent, identify active bleeding, and enable therapeutic embolization [3,20]. However, POCUS provides a rapid bedside alternative that is particularly useful in unstable patients requiring immediate triage and urgent clinical decision-making. In the present case, CT imaging was deferred because hemodynamic instability posed significant risks associated with transport to the radiology department. Additionally, preexisting renal dysfunction and potential risk of contrast-induced nephropathy favored an ultrasound-first approach. Ultrasonography is an inexpensive, readily available first-line imaging modality for evaluation of acute abdominal pain and can be useful for both the diagnosis and monitoring of RSH. However, ultrasound has a reported sensitivity of 80-90%; factors such as obesity, deep or complex hematomas, and operator dependence may impair its diagnostic accuracy [3,12]. The interpretation of ultrasound findings may be further complicated by variable echogenicity (hyperechoic, isoechoic, or anechoic appearance) across acute, subacute, and chronic stages of hematoma, as well as the presence of air foci or complex fluid collections, which can contribute to missed or delayed diagnoses [19]. These limitations may explain why the initial ultrasound in our patient failed to identify the evolving RSH.

Management of RSH depends on hematoma size, expansion, and hemodynamic stability. Initial management principles are similar to PPH management and include aggressive resuscitation, blood product replacement, and multidisciplinary coordination [1,2]. Most reported post-cesarean RSH cases required surgical intervention for evacuation of large hematoma, vascular ligation, and management for infected hematoma or intra-peritoneal rupture [6,8,12-14], whereas conservative management was successful in stable patients with smaller, nonexpanding hematomas [3,7,10,15-16]. Although transcatheter arterial embolization is an effective alternative [4], its use in obstetric RSH remains limited, particularly in unstable patients or those with renal dysfunction. In our patient, hemodynamic instability and impaired renal function precluded embolization, making surgical exploration essential for definitive diagnosis, hematoma evacuation, hemostasis, decompression, and drain placement.

Obstetric RSH, although rare, can be life-threatening. Historical reports described maternal mortality rates up to 13% and fetal mortality rates approaching 50% [8,10]; however, these estimates are based largely on older case reports and may not reflect contemporary outcomes. Despite advances in imaging and critical care, delayed diagnosis may still result in fatal complications, including multiorgan failure, renal failure, coagulopathy, and death [8,18]. Close post-treatment surveillance remains essential because rebleeding, infection, or coagulopathy may occur despite definitive management [7,8,10]. In our patient, serial laboratory and ultrasound monitoring after surgical evacuation showed stable recovery without further intervention.

While CT angiography was deferred due to hemodynamic instability and renal dysfunction, intraoperative findings revealed a large hematoma between the rectus sheath and transversalis fascia without intraperitoneal or prevesical extension, most consistent with type II RSH according to the Berna classification [21]. However, this classification was inferred intraoperatively and represents a limitation of the report. As a single case report, the study cannot establish causality or generalize diagnostic and therapeutic outcomes. Nevertheless, the case highlights important diagnostic and management challenges of severe post-cesarean RSH complicated by AKI and emphasizes the need for prospective evidence to guide management. Meticulous surgical technique, controlled retraction, and thorough hemostasis during cesarean delivery may help reduce the risk of epigastric vessel injury and subsequent RSH formation [5,9].

## Conclusions

To conclude, this case expands the sparse literature on post-cesarean RSH, particularly in severe preeclampsia complicated by hypovolemic shock and AKI requiring surgical evacuation and hemodialysis. RSH, while underemphasized in current PPH guidelines, represents a rare extrauterine and extragenital cause of concealed hemorrhage within the “trauma” component of the 4Ts framework. It should therefore be considered once uterine and genital tract causes have been excluded. Early suspicion, prompt imaging, and timely multidisciplinary management are essential, as RSH may mimic the typical presentation of PPH but requires a distinct diagnostic and therapeutic approach to reduce maternal morbidity and mortality.
